# ‘And breathe…’? The sociology of health and illness in COVID‐19 time

**DOI:** 10.1111/1467-9566.13110

**Published:** 2020-05-13

**Authors:** Catherine M. Will

**Affiliations:** ^1^ School of Law, Politics and Sociology University of Sussex Sussex UK

This note was written on 1^st^ April – April Fool's Day ‐ 2020 when we all vehemently wished that the global COVID‐19 pandemic could be forgotten as a bad joke. I put the date because at this point it feels that no one knows where we are going over the next few weeks and months, and it is curiously exposing to write in the centre of the pandemic. Typically a research note for our journal is meant to cover ‘a topical issue and/or an issue that has been neglected in some way, identifying research priorities’. It is not a form that has been much used in recent years, but I certainly do not think COVID‐19 is being or likely to be neglected. Nor do I primarily want to map out research priorities for other sociologists. Some of this is being done through blogs ‐ for example *Discover Society* 1 or *Cost of Living* 2 supported by the British Sociological Association *–* and sociologists have been prominent on social media offering rapid responses to the crisis from different angles. While many see in COVID‐19 evidence of the importance of solidarity or social protection, and threats to them from neoliberal practices, a huge swathe of other concerns and concepts in the sociology of health and illness also feel urgently relevant. However I want to start by insisting on the right not to rush to formulate new research or draw conclusions. We have chosen as a journal not to solicit new material at this relatively early stage of the COVID‐19 pandemic. No doubt numerous studies will be done but research can also take time. We suggest that our authors and readers ‘breathe’ when they can and research when it feels right.

Our current experience of the pandemic is all about breath. The spread of COVID‐19 has created risks in the simple act of breathing, and difficulties for those who suffer the virus badly. The paper that this note accompanies analyses some of the complexity of what the authors call ‘aerography’ for people at heightened risk before COVID‐19. Writing before this crisis, Brown, Buse, Lewis, Martin and Nettleton explore the challenges of minimising infection for people living with cystic fibrosis, and the ways these are made relevant for those designing, modifying and working within contemporary hospitals. As the authors suggest in a new introduction, these problems are now being experienced on a wider scale, as society faces the challenge of reducing transmission of an airborne virus that has spread across the world in a few months. Referencing previous discussions of ‘circuits of hygiene’ (Fox 1997), ‘hygienic prudence’ (Lowton and Gabe 2006) and ‘sterility as a product of spatial ordering’ (Mesman 2009) the authors develop their own theoretical palate for studies of illness transmission. The paper is a window on the lives of people for whom sensitivity to infection is nothing new, and who have lived with physical distancing and special hygiene measures for years. It is particularly interesting to me because it engages with the materialities and practices of preventing infection. This contrasts with much media coverage and sociological comment that has so far focussed more on discourse.

There are already many words written about the terms used to present the virus and there will no doubt be more. In just one week of UK lockdown there seems to have been a shift away from the military metaphors which are familiar in medical sociology (Nerlich and James 2009, Sontag 1989) towards comparing COVID‐19 to natural disasters such as earthquakes and tsunamis. Researchers have drawn attention to the ways in which nationalism and racism are also shaping responses, for example in Meinhof's (2020) discussion of the sinophobia, new orientalism and colonial thinking inscribed into accounts of the early experience of the Wuhan region in China. The established literature on the ways in which we frame disease can offer useful comparisons for accounts of the COVID‐19 event, including work on other epidemics such as the 1918 Influenza outbreak, Ebola and outbreaks of avian and other flu (e.g. Staniland and Smith 2013 in this journal's own special issue on pandemics published in 2013). However we should also look at what people do. Ordinary people are part of a hasty mass movement for developing lay virologies and epidemiologies (after Davison *et al*. 1991) relevant to COVID‐19. New and revived practices include thorough handwashing, disinfection, covering the mouth and nose, physical distancing, self‐isolation or quarantine and close attention to signs of the presence and severity of the disease. Crowd sourcing studies of different kinds are also starting to gather this information, which finds an audience in part because clinical testing has not been easy to access in many countries.

Other practices and tools informing government actions have also attracted attention. Last week Rhodes *et al*. (2020) published a paper arguing that across the world much of the discussion of COVID‐19 has been conducted around mathematical models and modelling experts ‐ a dynamic also discussed by Mansnerus (2013) for earlier pandemics. These authors emphasised pressures to reduce the distance between experts and the public, suggesting that ‘people want to input, to make and translate [COVID‐19] evidence, not merely receive [it]’. Sociologists have taken issue with the apparent influence of ‘behavioural economics’ in UK policy. Both Bacevic (2020) and McGoey (2020) have critiqued the use of ‘nudge’ ideas in attempting to predict and manipulate people's actions, insisting on people's capacity for adaption, reflection and social organisation. Different writers have called for clearer efforts to involve the public in policymaking (Kearnes *et al*. 2020, Pieri 2020). The tendency to restrict movement within and across the borders of nation states, and efforts to surveil and exclude specific groups, show the familiar moves by which populations are imagined through the lens of race and with reference to geo‐political borders (Hoffman 2013, Kehr 2012, Taylor 2013).

Other kinds of politics have been encountered as activists as much as scholars. Sociologists who have long worked with advocates for marginalised groups have joined debates about the difficulties for people in precarious work or housing observing physical distancing policies, explaining how they may struggle to avoid infection through hygiene measures or to access care in hospitals under pressure. One example of success with this comes in National Institute of Health and Care Excellence (NICE) guidelines on access to critical care (NICE 2020a). These attempted to supplement the use of age as a prioritisation principle ‐ used alone in some European countries to decide how to allocate scarce ventilators ‐ with a measure of frailty. Numerous people rushed to point out that this meant potentially denying treatment to people living with stable physical and mental impairments and the guidelines were amended in a few days (NICE 2020b). Yet debates that were previously pursued at a national level through institutions like NICE are also being played out as local dramas around hospital admission and the allocation of patients to beds. These have included questions about whether residents of social care homes will be transferred or resuscitated if suffering severe illness. Rationing is also taking place through hasty efforts at prioritisation of COVID‐19 against the other work that already filled hospitals – oncology, obstetrics, neurology and the rest. In these efforts different healthcare professionals are being asked to bear new responsibility in addition to the manifest risks of interacting with potential COVID‐19 patients (see work on these moral burdens in the case of Ebola by Broom and Broom 2017). All of this is familiar ground for readers of this journal and will be important sites for research in medical sociology. At the same time the involvement of different professions and organisations in enforcing and elaborating governmental responses, including the police and military, may require broader engagement with other sociological and criminological traditions. Studies of public health disasters also encourage analysis of the ways in which the roles of government, voluntary and media organisations are disrupted and evolve at such times (e.g. Klinenberg 2002, Treichler 1999). Comparisons can again be found in the pandemic special issue of this journal (e.g. French and Mykhalovskiy 2013, Gislason 2013).

In addition to studies of the politics of public health interventions and healthcare delivery, we will no doubt want to do in‐depth investigations of the experience of healthcare staff, of other essential workers and of patients or potential patients. One important issue is the extent to which healthcare professionals are being asked to work outside their specialism, in new hierarchies and with new digital and bio‐technologies. Families of patients are facing distressing restrictions on their caring involvement due to infection risk. Narratives about these experiences are appearing through social media, and sociologists of digital practices have started to comment on these (Halford 2020 2020, Lupton 2020). Though there are already fertile links between scholarship in the sociology of health and illness and critical data studies or new media studies, I hope further exchange will develop from the experience of COVID‐19, drawing explicitly on existing ways of understanding illness narratives and sense‐making. As a discipline sociology has long been attentive to forms of exclusion and marginalisation in digital interactions as well as the potential for collective mobilisation by patients and their advocates, and has much to offer.

This is only a sketch of fruitful directions, scarcely a map. I hope that future research in the sociology of health and illness will draw on the established strengths of our field but be open to other fields in sociology and beyond, capitalising on new interest in health and illness. This work can be grounded in shared questions about government intervention and relationships with citizens and those excluded from that category; diverse forms of inequality and marginality; the practices of living with risk for individuals, families and communities in different sites and scales; and thus offer analyses of ableism, ageism, racism and nationalism as developed through the COVID‐19 pandemic. While all these feel relevant in the context of the UK, researchers should resist the occasional temptation to think of the UK and the National Health Service as necessarily different, seeking to think comparatively and draw on analysis of different countries’ experiences as well as global health institutions, actors and practices. This should include being ready to engage with work in social anthropology, international relations, science and technology studies and newer fields that are important in the growing study of global health.

One final example of openness to new concepts and topics can be found in the Brown *et al*. paper, which was my impetus for writing. Many classic sociological studies of hospitals made little of their material architecture and spatial organisation, as they were conceptualised primarily as social institutions, defined through professional groups working in hierarchies with their own forms of action. In this paper the authors show how architectural conventions for hospitals evolved, and today enable and restrict responses to infection risk. Such analysis may be vital in a situation where new treatment centres are being thrown up in a matter of days and existing ones radically reconfigured. Meanwhile at a smaller scale the kind of ‘total atmospheric immunisation’ viewed impossible by one of their respondents, a respiratory doctor, is being reached towards if still not practised as people to struggle to stay safe while treating patients in these ‘hospitals’. Institutions like care homes, prisons and immigration centres are being revealed as hugely unsafe places in which the virus can spread rapidly and in the case of care homes may not be met with much medical intervention.

All these issues are being explored and debated as I write, and no doubt more forms of exclusion and disruption will be identified by the time this is published. I hope very much that things look somewhat better by then. Yet I also anticipate reading rich accounts of the coronavirus pandemic in due course that show sociology's commitment to understanding marginalisation in all its forms and the connections between government practices and actions and the experience of the virus across Europe and in the rest of the world.

## Author contributions

Catherine M. Will was solely responsible for the conceptualization, investigation and writing of this note.

## Notes

1 See website at: https://discoversociety.org/category/quickfire/covid-19/.

2 See website at https://www.cost-ofliving.net.
